# Lessons on malaria vector control from Bashful, Doc, Dopey, Grumpy, Happy, Sneezy, and Sleepy

**DOI:** 10.5281/zenodo.10907072

**Published:** 2024-04-02

**Authors:** Manuel F. Lluberas

**Affiliations:** 1 Mosquito Den LLC, Río Grande, Puerto Rico.

## Abstract

Despite significant advancements in vector control, malaria continues to expand and claim hundreds of thousands of lives annually. A 1943 animated film by Walt Disney remains a poignant reminder of the ongoing challenge and a good example of interventions that have fallen off the pages of history. It underscores two key points. First, the importance of proactive mosquito control measures and the need for comprehensive strategies targeting mosquitoes at every stage of their life cycle. Second, collaboration between all stakeholders and sustained investment are vital for success in malaria control. Manuel Llu-beras is a public health entomologist renowned globally for assembling the business architecture of mosquito population management initiatives in four continents. He crafted the first WHO Operational Manual for Indoor Residual Spraying (IRS) and played a pivotal role in designing the structure of the IRS campaign of the US President’s Malaria Initiative and several mineral extraction companies. He served in several post-event emergency mosquito control operations. Prior to establishing Mosquito Den LLC in 2021, he was Executive Director for Public Health for H.D. Hudson Manufacturing from 1996 through 2022. He served as medical entomologist for the US Navy a dozen years. His contributions to public health entomology were recognised with the Global Trade Award from the Global Trade Chamber, the Meritorious Service Award of the American Mosquito Control Association, and two nominations for the Rear Admiral Charles S. Stevenson Award for excellence in US Navy Preventive Medicine.

In the annals of history, there are moments that transcend their time and place, leaving an indelible mark on the collective consciousness. One such moment occurred in 1943, with the release of Walt Disney's animated short film, *The Winged Scourge*.

Produced eighty years ago in collaboration with the now-defunct US Government Agency ‘Office of the Coordinator of Inter-American Affairs’, it sought to raise awareness about the threat of malaria in the United States and the importance of implementing active mosquito control measures to control it.

Fast forward eight decades, the lessons provided by the ‘Seven Magical Creatures’ in *The Winged Scourge* remain as relevant as ever but have fallen off the pages of history even as we confront the persistent challenges of a continuing expansion of malaria around the globe. It is imperative that we revisit the insights offered by this timeless classic [1].

At its core, *The Winged Scourge* serves as a stark reminder of the devastating toll that malaria exacts on communities. Through vivid animation and poignant narration, the film illustrates the life cycle of the malaria parasite and the role of mosquitoes as its vector and underscores the urgency of implementing comprehensive integrated mosquito control measures to mitigate its spread.

*The Winged Scourge* video was produced in 1943! Today’s programme managers have tools like portable calculators, computers, mobile phones, satellite imagery, drones, and artificial intelligence capable of identifying mosquitoes using a mobile phone. Current technology helps us identify mosquito sources from a satellite, link that information to a portable phone or computer, and use it to direct the pin-point application of target-specific and environmentally sound vector control materials from a drone. These were not even science fiction dreams when Disney’s video was produced!

The steps depicted in the video (Figure 1) must be adopted and adapted by all agencies and organisations working on or promoting global malaria vector control. Regrettably, these tools are not considered valid by many of those who decide what mosquito population suppression measure to deploy, or the direction malaria vector control programmes should take. Sadly, this video and the methods illustrated in it have suffered the same fate as the documented victories against malaria vectors obtained by Fred Soper, Israel Kligler, William Gor-gas, and several other ladies and gentlemen who controlled or eradicated malaria and other mosquito-borne diseases from over one hundred countries before the end of the Twentieth Century and remain in anonymity today.

**Figure 1.**
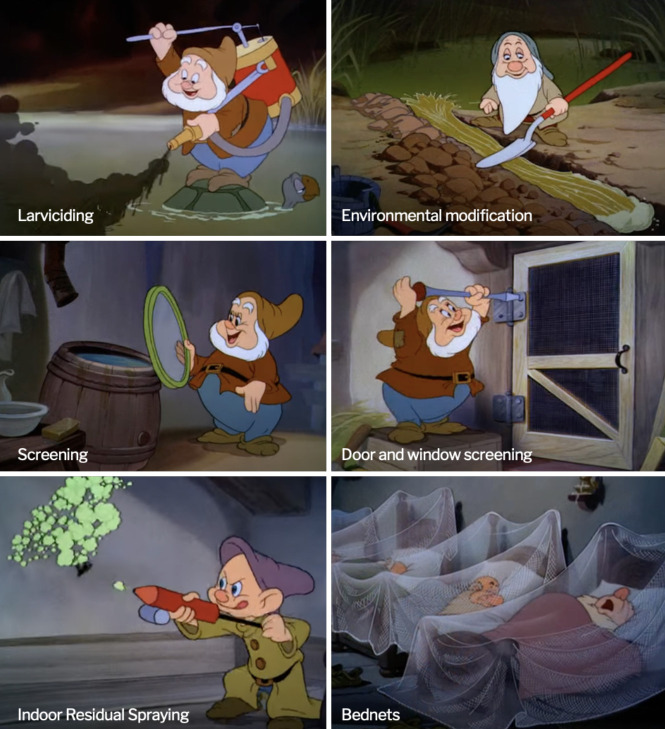
Stills from the Walt Disney movie *The Winged Scourge*, released in 1943. Although Indoor Residual Spraying and insecticide-treated bednets are still in use, other methods that played a crucial role in eliminating malaria from many countries, like larval source management (LSM) or house improvement, have largely been forgotten and should be revived.

Consequently, despite the countless advancements in science and technology, many malaria control programmes have failed to achieve their intended objectives. Malaria vector control demands everyone’s attention. The sobering reality is that malaria claims hundreds of thousands of lives annually and forces those who carry the malaria yoke to accept the inevitable fact that they will have to continue bearing its burden into the foreseeable future.

So, where have we gone wrong? What lessons can we glean from *The Winged Scourge* to inform our approach to malaria control in the modern era? First and foremost, we must recognise the importance of *proactive*, rather than *reactive*, measures in combating malaria and other vector-borne diseases. *The Winged Scourge* emphasises the need for comprehensive integrated vector management strategies that target mosquitoes at every stage of their life cycle. This includes efforts to eliminate their sources, deploying larval source modification and larviciding, controlling adult mosquitoes, and educating communities about the importance of personal protection measures and community-based interventions.

Yet, global malaria control programmes today continue to fall short in implementing these critical interventions. Too much emphasis has been placed on reactive and passive measures such as the distribution of insecticide-treated bednets (LLINs) and Indoor Residual Spraying (IRS) while neglecting the fundamental importance of active mosquito population suppression.

*The Winged Scourge* highlights the need for collaboration and coordination among all stakeholders in the fight against malaria. We see in the film how government agencies, healthcare workers, and community members come together to implement mosquito control measures effectively. This spirit of collective action is as relevant today as it was in 1943!

In many regions, fragmented approaches and competing interests have hindered progress in malaria control efforts. Bilateral funders, governments, non-governmental organisations, and other stakeholders must work together in a coordinated manner to align resources, share best practices, and implement evidence-based active interventions.

*The Winged Scourge* underscores the importance of sustained investment in malaria control programmes. In the film, we witness the dedication and perseverance of those involved in mosquito control efforts, despite facing numerous challenges along the way. It serves as a powerful reminder that progress in malaria control requires long-term commitment and support.

Inexplicably, however, many malaria control programmes have been plagued by inconsistent funding, shifting priorities, and donor fatigue. This lack of sustained investment has hindered efforts to scale up interventions, strengthen health systems, and build resilience against malaria.

*The Winged Scourge* offers valuable insights into the challenges and opportunities of malaria control. As we confront the enduring threat of malaria and other vector-borne diseases, we must heed the lessons presented by these ‘Seven Magical Creatures’ and redouble our efforts to implement comprehensive, proactive, and sustainable mosquito control measures. Only then can we hope to turn the tide in the fight against malaria and ensure a healthier, more prosperous future for all.

Let us draw inspiration from its timeless message and recommit ourselves to the task of malaria vector control with renewed vigour and determination. Together, we can overcome the scourge of malaria and other vector-borne diseases and build a world where no one lives in fear of this deadly disease.

Happy hunting.
